# Clinical factors associated with adherence to aerobic and resistance physical activity guidelines among cancer prevention patients and survivors

**DOI:** 10.1371/journal.pone.0220814

**Published:** 2019-08-01

**Authors:** Adriana M. Coletta, Guillermo Marquez, Parijatham Thomas, Whittney Thoman, Therese Bevers, Abenaa M. Brewster, Ernest Hawk, Karen Basen-Engquist, Susan C. Gilchrist

**Affiliations:** 1 Cancer Control and Population Sciences Program, Huntsman Cancer Institute, Salt Lake City, Utah, United States of America; 2 Department of Health, Kinesiology, and Recreation, The University of Utah, Salt Lake City, Utah, United States of America; 3 Department of Clinical Cancer Prevention, The University of Texas MD Anderson Cancer Center, Houston, Texas, United States of America; 4 Division of OVP, Cancer Prevention and Population Sciences, The University of Texas MD Anderson Cancer Center, Houston, Texas, United States of America; 5 Department of Behavioral Science, The University of Texas MD Anderson Cancer Center, Houston, Texas, United States of America; 6 Department of Cardiology, The University of Texas MD Anderson Cancer Center, Houston, Texas, United States of America; University of Nebraska Medical Center, UNITED STATES

## Abstract

Physical activity (PA) is a known behavior to reduce cancer risk and improve cancer survivorship, yet adherence to PA guidelines is poor among the general population and cancer survivors. The purpose of this study was to determine the extent to which patients referred for exercise consultation within a clinical cancer prevention setting were meeting aerobic and resistance physical activity (PA) guidelines and to identify factors associated with guideline adherence. Between 2013 and 2015, cancer prevention patients and cancer survivors were interviewed by an exercise physiologist within an Integrative Health Program at The University of Texas MD Anderson Cancer Prevention Center. PA adherence was defined as at least 150-minutes of moderate-intensity or 75-minutes of vigorous-intensity PA per week, along with resistance training at least 2 days per week. Logistic regression was used to determine factors associated with meeting or not meeting PA guidelines for aerobic exercise, resistance exercise, and aerobic and resistance exercise combined. Among 1,024 cancer prevention patients and survivors, 9% of patients adhered to guideline-based PA. Adherence to aerobic and resistance guidelines were 20% and 12%, respectively. Overweight or obesity was associated with not meeting guideline-based PA in both cancer prevention patients and cancer survivors. Among breast cancer survivors, combination treatment with surgery, radiation, and chemotherapy (‘multimodal therapy’) was robustly associated with not meeting aerobic guidelines (OR 2.20, 95% CI: 1.17 to 4.16). BMI and breast cancer treatment history are key determinants of PA behavior among cancer prevention patients and survivors. Poor adherence to PA guidelines is a key issue for cancer prevention patients and survivors, particularly obese patients and women who receive multimodal therapy for breast cancer. Identifying and connecting patients at highest risk of poor PA adherence with exercise programs is needed to improve PA, a key modifiable cancer risk factor.

## Introduction

Physical activity is a known behavior to reduce cancer risk [1–8]. Moreover, among cancer survivors, engaging in physical activity is associated not only with improvements in survival [9–12], but also with improvements in quality of life [13, 14], body image, emotional well-being, sleep, sexuality [14], muscular strength and cardiorespiratory fitness [13], and reductions in fatigue [13, 14], depression [13], and pain [14]. Among the lifestyle guidelines linked with cancer prevention (i.e. physical activity, diet, weight management), adherence to physical activity guidelines has exhibited the strongest association with lower mortality rates in cancer survivors [10, 15]. As such, national guidelines are in place and promote 150 minutes of moderate-intensity or 75 minutes of vigorous-intensity physical activity per week, along with resistance training 2–3 days per week [16].

Significant reductions in cancer risk and improved survival has been demonstrated among those who adhere to the physical activity guidelines [17–19]. For example, a prospective study conducted in nearly 500,000 men and women in the United States observed a significant inverse association (p<0.05) between adherence to aerobic physical activity guidelines and both cancer incidence and cancer-specific mortality [18]. In addition, in a retrospective cohort based on the National Institute of Health AARP Diet and Health Study, conducted among 215,122 men and women, participation in resistance training was significantly associated with a lower risk of colon cancer (p-trend = 0.003) and trend towards lower risk of kidney cancer (p-trend = 0.06) [20]. Further, a retrospective study based on the National Health Interview Survey, conducted among nearly 14,000 cancer survivors at least three-years post-treatment, observed that adherence to both aerobic and resistance physical activity guidelines was associated with a 48%, 46%, and 40% reduction in cancer-specific, cardiovascular disease-specific, and all-cause mortality, respectively [19]. This work supports the importance of promoting physical activity in both cancer prevention and survivorship.

Unfortunately, population-based studies have shown that adherence to the physical activity guidelines is generally poor in both the general population and cancer survivors. According to surveillance data from the Centers for Disease Control and Prevention, approximately 20% of US adults are meeting both aerobic and resistance physical activity guidelines [4]. Among cancer survivors, 9–20% meet both aerobic and resistance physical activity guidelines [19, 21–26], with 22–44% only meeting aerobic guidelines [24, 25] and about 10–34% only meeting resistance guidelines [24, 25, 27, 28]. Evidence suggests that physical activity counseling from a health care provider improves physical activity behaviors, both among cancer survivors [29–31] and the general population [31]. However, initial factors that associate poor adherence to guideline-based physical activity before counseling is not well understood, though of key importance when developing and promoting initiatives to help patients reach recommendations.

At the University of Texas MD Anderson Cancer Prevention Center, patients with and without a history of cancer are seen for preventive services. The primary aim of the present investigation was to determine the extent to which cancer prevention patients and survivors seen in this setting were meeting aerobic physical activity guidelines, resistance physical activity guidelines, and the combination of aerobic and resistance physical activity guidelines. Our secondary aim was to identify clinical factors independently of physical activity guideline adherence among cancer survivors.

## Methods

### Design, patient population, and exercise counseling

A retrospective chart review was conducted from patients that were referred for consultation from an exercise physiologist between 2013 and 2015 in the Cancer Prevention Center at MD Anderson Cancer Center. The protocol and waiver of informed consent was approved by The University of Texas MD Anderson Cancer Center Institutional Review Board.

The patient population seen at MD Anderson’s Cancer Prevention Center (CPC) consists of patients of average to high risk for cancer (cancer prevention patients) and cancer survivors who have completed treatment. Cancer prevention patients of average risk may be representative of the general population, whereas higher risk prevention patients are unique to this setting.

The CPC offers a range of services to help patients (prevention patients and survivors) learn how to reduce cancer risk or to detect cancer early. The CPC implemented an integrative health program that assists patients in adopting healthy lifestyle behaviors to prevent cancer occurrence or recurrence. Included in this program is the opportunity for in-depth one-on-one exercise counseling with a clinical exercise physiologist. When patients arrive for their clinic appointment, they are asked if they would like the opportunity to meet with a clinical exercise physiologist for exercise counseling. Additionally, patients can be referred to the exercise physiologist for exercise counseling by their provider if the need or interest for exercise counseling is identified by the patient or provider. Providers in the CPC consist of internists/family physicians, medical oncologists, and advance nurse practitioners.

Within the exercise counseling sessions, our clinical exercise physiologists work with our patients to identify their personal goals related to increasing physical activity behavior and strategize a personalized exercise plan to help them achieve their goals. The degree of follow-up and mode of follow-up (e.g.- phone call or face-to-face appointment) is individualized based on patient preference. Data regarding adherence to both aerobic and resistance guidelines are collected during these counseling sessions (please see assessment of adherence to physical activity guidelines section for details).

Between 2013 and 2015, a total of 10,000 patients were seen by the integrative health program. During this time exercise physiologist interviewed a total of 1,126 patients. We limited the analysis to patient charts including information on aerobic activity and resistance training (n = 1,035). We then eliminated patients with a BMI < 18.5 kg/m^2^ (n = 4) and patients with an undetermined race/ethnicity (n = 7) due to inadequate sample size in these cells for analyses. Therefore, a total of 1,024 cancer prevention patients and cancer survivors were included in this investigation.

### Patient demographics and clinical factors

Information regarding the following demographic and clinical factors was collected from the medical record: age, sex, race/ethnicity, body mass index (BMI), smoking history, cancer history, diabetes status and menopause status. For the sample of cancer survivors, time since diagnosis and cancer therapy history (i.e. history of chemotherapy, anthracycline therapy, radiation therapy, radiation side, surgery, surgery type) were also collected. Multimodal therapy was defined as patients undergoing all three types of therapy (chemotherapy, radiation, surgery) as part of their treatment plan.

### Assessment of adherence to physical activity guidelines

Current self-reported physical activity levels and adherence to the physical activity guidelines were determined during the exercise physiologist consultation. Patients were asked the following questions within a conversation pertaining to their exercise behavior: how many days per week do you perform moderate exercise; on average, how long do you perform these exercises; how many days per week do you perform vigorous exercises; on average, how long do you perform these exercises; how many days per week do you practice muscle strengthening exercises; on average, how long do you perform these exercises; what is the mode of activity you typically engage in; what are the exercise resources available to you (i.e. gym, fitness classes, exercise videos). Patients were also provided information defining what is considered moderate intensity and vigorous intensity aerobic activity, and resistance training. Patients were considered adherent to the aerobic physical activity guidelines if they reported participating in at least 150 minutes of moderate intensity aerobic activity per week, or at least 75 minutes of vigorous intensity aerobic activity per week, or a combination of both. Patients were considered adherent to the resistance training guidelines if they reported participating in at least two days of resistance training per week that worked all major muscle groups. Patients were considered adherent to both aerobic and resistance training guidelines if they reported participating in the aforementioned amount of weekly aerobic and resistance training activity.

### Statistical methods

The variables pertaining to meeting aerobic, resistance, and the combination of aerobic and resistance guidelines were dichotomized as “meeting” or “not meeting” the guidelines. Frequencies and descriptive statistics were used to determine patient characteristics and the extent of which patients were adherent to aerobic, resistance, and the combination of aerobic and resistance guidelines. Chi-square tests were used to examine if there was a significant association between adherence to aerobic guidelines, resistance guidelines, and the combination of aerobic and resistance guidelines, by demographic and clinical factors among the total sample (n = 1,024) and by treatment exposure among female breast cancer survivors (n = 346). For the cancer survivor analyses, female breast cancer was the cancer type used in the analysis due to the small sample size and heterogeneity of cancer types other than female breast cancer. The other most common types of cancer (>1%) seen in the Cancer Prevention Center included: skin cancers other than melanoma (7%, n = 36), thyroid cancer (6%, n = 30), melanoma (3%, n = 15), colon cancer (2%, n = 12), and endometrial cancer (2%, n = 9).

Univariate logistic regression modeling was performed to determine the relative odds of not meeting aerobic physical activity guidelines (yes/no) based on individual baseline demographics and clinical factors for the overall CPC population (n = 1,024) and breast cancer survivors (n = 346). Similar modeling was performed for not meeting resistance guidelines only (yes/no) and not meeting both aerobic and resistance physical activity guidelines (yes/no). Multivariable models adjusted for age, BMI, race/ethnicity, insurance status, smoking history, and diabetes status. Additional adjustments were made for menopause status and multimodal breast cancer therapy for breast cancer specific models. All data were analyzed with SPSS statistical software package, version 23 (Chicago, IL).

## Results

### Cancer prevention patients and survivors referred to an exercise physiologist

#### Demographic and clinical factors

Of the 1,024 patients included in the present study, 97% (n = 989) were women and 3% (n = 35) were men. The mean age of participants was 57±10 years old. Sixty-two percent (63%) were Caucasian (n = 642), 21% were African-American (n = 217), 12% were Hispanic (n = 125), and 4% were Asian (n = 40). The mean BMI was 33.4±7.1 kg/m^2^. A total of 9% of patients were normal weight (BMI 18.5–24.9 kg/m^2^, n = 94), 24% were overweight (BMI 25–29.9 kg/m^2^, n = 245), and 67% were obese (BMI ≥30 kg/m^2^, n = 685). A total of 76% (n = 778) of patients were never smokers, and 21% and 3% were former (n = 215) or current (n = 31) smokers respectively. A total of 83% did not have a history of diabetes (n = 852), while 3% had pre-diabetes (n = 29), and 14% had diabetes (n = 143). Finally, among women, 18% were pre-menopausal (n = 179), 2% were peri-menopausal (n = 22), and 80% were post-menopausal (n = 788). Fifty percent of patients (n = 513) were cancer prevention patients and 50% were cancer survivors (n = 511).

Among breast cancer survivors, mean age and BMI of participants was 61±10 years old and 32±7 kg/m^2^. A total of 15% of patients were normal weight (BMI 18.5–24.9 kg/m2, n = 53), 25% were overweight (BMI 25–29.9 kg/m2, n = 85), and 60% were obese (BMI ≥30 kg/m2, n = 208). A total of 10% were pre-menopausal (n = 35), 2% were peri-menopausal (n = 7), and 88% were post-menopausal (n = 304). Sixty percent (60%) were Caucasian (n = 207), 20% were African-American (n = 70), 15% were Hispanic (n = 51), and 5% were Asian (n = 18). A total of 75% (n = 260) of patients were never smokers, 23% (m = 78) were former smokers and 2% (n = 8) were current smokers. A total of 82% did not have a history of diabetes (n = 285), while 3% had pre-diabetes (n = 9), and 15% had diabetes (n = 52).

#### Adherence to physical activity guidelines by demographic and clinical factors

Among our cohort 20% met aerobic physical activity guidelines. Men were more likely to meet aerobic guidelines compared to women (34% compared to 20%, p = 0.03) (Table 1). Individuals with a weight classification of obesity were less likely to meet aerobic physical activity guidelines compared to overweight or normal weight individuals (16% compared to 26% and 35%, respectively, p<0.001). Individuals with a cancer history were more likely to meet aerobic physical activity guidelines compared to patients with no cancer history (23% compared to 17%, p = 0.02). Univariate associations were not observed for age (p = 0.07), race/ethnicity (p = 0.10), smoking history (p = 0.82), history of diabetes (p = 0.06), or menopausal status (p = 0.61). Regarding resistance guidelines, 12% of cancer prevention patients and survivors met recommended guidelines. Both BMI (p<0.001) and cancer history (p = 0.04) were associated with adherence to resistance guidelines, where individuals with normal BMI or cancer survivors were more likely to meet guidelines. Moreover, a significant association was also observed for age, such that middle-aged (45–64 years old) individuals were more likely to meet resistance physical activity guidelines (p = 0.01). No other univariate associations were observed regarding adherence to resistance guidelines (p>0.05).

**Table 1:** Adherence to physical activity guidelines by demographic and clinical factors.

Variable			Aerobic Guidelines			Resistance Guidelines			Aerobic & Resistance Guidelines		
			Met	Not Met	p-level	Met	Not Met	p-level	Met	Not Met	p-level
Age (years)	< 45	Count	14	102	0.07	6	110	**0.01**	4	112	0.07
	(n = 116)	%	12.1%	87.9%		5.2%	94.8%		3.4%	96.6%	
	45–54	Count	56	204		41	219		29	231	
	(n = 260)	%	21.5%	78.5%		15.8%	84.2%		11.2%	88.8%	
	55–64	Count	92	308		57	343		40	360	
	(n = 400)	%	23.0%	77.0%		14.3%	85.8%		10.0%	90.0%	
	65–74	Count	39	169		21	187		14	194	
	(n = 208)	%	18.8%	81.3%		10%	90%		6.7%	93.3%	
	≥ 75	Count	5	35		2	38		2	38	
	(n = 40)	%	12.5%	87.5%		5.0%	95.0%		5.0%	95.0%	
Sex	Female	Count	194	795	**0.03**	119	870	0.06	83	906	0.07
	(n = 989)	%	19.6%	80.4%		12.0%	88.0%		8.4%	91.6%	
	Male	Count	12	23		8	27		6	29	
	(n = 35)	%	34.3%	65.7%		22.9%	77.1%		17.1%	82.9%	
Race/ Ethnicity	White (non-Hispanic)	Count	142	500	0.10	89	553	0.12	64	578	0.29
	(n = 642)	%	22.1%	77.9%		13.9%	86.1%		10.0%	90.0%	
	Asian	Count	10	30		7	33		3	37	
	(n = 40)	%	25.0%	75.0%		17.5%	82.5%		7.5%	92.5%	
	African American	Count	35	182		19	198		13	204	
	(n = 217)	%	16.1%	83.9%		8.8%	91.2%		6.0%	94.0%	
	Hispanic or Latino	Count	19	106		12	113		9	116	
	(n = 125)	%	15.2%	84.8%		9.6%	90.4%		7.2%	92.8%	
BMI (kg/m^2^)	18.5–24.99	Count	33	61	**<0.001**	21	73	**<0.001**	16	78	**<0.001**
	(n = 94)	%	35.1%	64.9%		22.3%	77.7%		17.0%	83.0%	
	25–29.99	Count	63	182		41	204		30	215	
	(n = 245)	%	25.7%	74.3%		16.7%	83.3%		12.2%	87.8%	
	≥ 30	Count	110	575		65	620		43	642	
	(n = 685)	%	16.1%	83.9%		9.5%	90.5%		6.3%	93.7%	
Smoking History	Never	Count	159	619	0.82	89	689	0.23	65	713	0.79
	(n = 778)	%	20.4%	79.6%		11.4%	88.6%		8.4%	91.6%	
	Former	Count	42	173		34	181		21	194	
	(n = 215)	%	19.5%	80.5%		15.8%	84.2%		9.8%	90.2%	
	Current	Count	5	26		4	27		3	28	
	(n = 31)	%	16.1%	83.9%		12.9%	87.1%		9.7%	90.3%	
Cancer History	No	Count	88	425	**0.02**	53	460	**0.04**	36	477	0.06
	(n = 513)	%	17.2%	82.8%		10.3%	89.7%		7.0%	93.0%	
	Yes	Count	118	393		74	437		53	458	
	(n = 511)	%	23.1%	76.9%		14.5%	85.5%		10.4%	89.6%	
Diabetes	No	Count	183	671	0.06	113	741	0.19	82	772	0.07
	(n = 854)	%	21.4%	78.6%		13.2%	86.8%		9.6%	90.4%	
	Pre-diabetes	Count	4	23		2	25		1	26	
	(n = 27)	%	14.8%	85.2%		7.4%	92.6%		3.7%	96.3%	
	Yes	Count	19	124		12	131		6	137	
	(n = 143)	%	13.3%	86.7%		8.4%	91.6%		4.2%	95.8%	
Menopause (n = 989)	Pre-menopausal	Count	33	146	0.61	21	158	0.08	17	162	**0.04**
	(n = 179)	%	18.4%	81.6%		11.7%	88.3%		9.5%	90.5%	
	Peri-menopausal	Count	6	16		6	16		5	17	
	(n = 22)	%	27.3%	72.7%		27.3%	72.7%		22.7%	77.3%	
	Post-menopausal	Count	155	633		92	696		61	727	
	(n = 788)	%	19.7%	80.3%		11.7%	88.3%		7.7%	92.3%	

**Fig 1:**
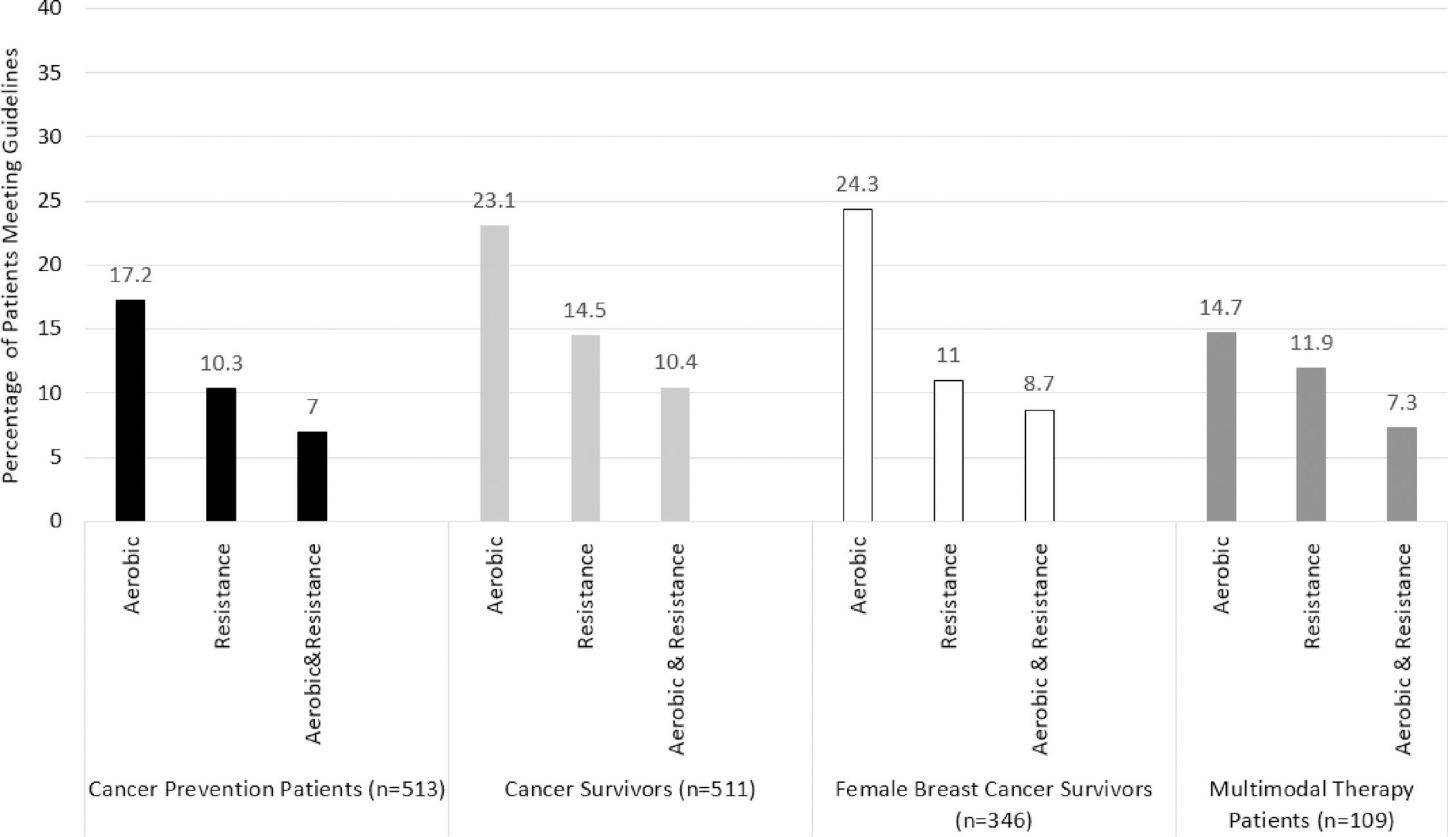
Percentage of patients meeting physical activity guidelines.

Overall, 9% of cancer prevention patients and survivors met both aerobic and resistance physical activity guidelines. Only 6% of obese individuals met both guidelines, whereas 12% of overweight and 17% of normal weight met both guidelines, respectively (p<0.001). Among women, a significant association was observed for menopausal status, where peri-menopausal women were most-likely to meet both guidelines (22.7%) followed by pre-menopausal (9.5%) then post-menopausal women (7.7%) (p = 0.04). No other significant associations were observed (p>0.05 for all) (Fig 1).

### Female breast cancer survivors

The mean time from diagnosis to physical activity assessment was 9.1 years (95% confidence interval [CI] 8.5–9.7). Among female breast cancer survivors, there was no association between meeting aerobic physical activity guidelines and time since cancer diagnosis (p = 0.53), history of chemotherapy (p = 0.19), anthracycline therapy (p = 0.61), or surgery (p = 0.13). Univariable associations were observed for radiation therapy, such that women who did not undergo radiation therapy were more likely to meet aerobic activity guidelines (31%) compared to women who did have radiation therapy (20%, p = 0.02). Additionally, women who had a mastectomy were more likely to meet aerobic activity guidelines compared to women who have had lumpectomy, 32% compared to 20% respectively (p = 0.01). Associations between all other variables in relation to resistance training guidelines alone and both aerobic and resistance guidelines are seen in Table 2.

**Table 2:** Adherence to physical activity guidelines by treatment exposure among female breast cancer survivors.

Variable			Aerobic Guidelines			Resistance Guidelines			Aerobic & Resistance Guidelines		
			Meeting	Not Meeting	p-level	Meeting	Not Meeting	p-level	Meeting	Not Meeting	p-level
Time Since Cancer Diagnosis (n = 346)	≤ 3 years	Count	12	45	0.53	8	49	0.98	6	51	0.90
	(n = 57)	%	21.1%	78.9%		14.0%	86.0%		10.5%	89.5%	
	> 3 years	Count	72	217		41	248		32	257	
	(n = 289)	%	24.9%	75.1%		14.2%	85.8%		11.1%	88.9%	
Chemotherapy (n = 346)	No	Count	52	141	0.19	29	164	0.60	24	169	0.33
	(n = 193)	%	26.9%	73.1%		15.0%	85.0%		12.4%	87.6%	
	Yes	Count	32	121		20	133		14	139	
	(n = 153)	%	20.9%	79.1%		13.1%	86.9%		9.2%	90.8%	
Anthracycline Therapy (n = 153)	No	Count	2	5	0.61	2	5	0.21	1	6	0.63
	(n = 7)	%	28.6%	71.4%		28.6%	71.4%		14.3%	85.7%	
	Yes	Count	30	116		18	128		13	133	
	(n = 146)	%	20.5%	79.5%		12.3%	87.7%		8.9%	91.1%	
Radiation Therapy (n = 346)	No	Count	42	94	**0.02**	20	116	0.82	17	119	0.47
	(n = 136)	%	30.9%	69.1%		14.7%	85.3%		12.5%	87.5%	
	Yes	Count	42	168		29	181		21	189	
	(n = 210)	%	20.0%	80.0%		13.8%	86.2%		10.0%	90.0%	
Radiation Side (n = 210)	Left	Count	22	88	1.00	14	96	0.63	8	102	0.17
	(n = 110)	%	20.0%	80.0%		12.7%	87.3%		7.3%	92.7%	
	Right	Count	20	80		15	85		13	87	
	(n = 100)	%	20.0%	80.0%		15.0%	85.0%		13.0%	87.0%	
Surgery (n = 346)	No	Count	0	7	0.13	0	7	0.28	0	7	0.35
	(n = 7)	%	0.0%	100.0%		0.0%	100.0%		0.0%	100.0%	
	Yes	Count	84	255		49	290		38	301	
	(n = 339)	%	24.8%	75.2%		14.5%	85.5%		11.2%	88.8%	
Surgery Type (n = 339)	Mastectomy	Count	46	100	**0.01**	27	119	0.07	23	123	**0.02**
	(n = 146)	%	31.5%	68.5%		18.5%	81.5%		15.8%	84.2%	
	Lumpectomy	Count	38	155		22	171		15	178	
	(n = 193)	%	19.7%	80.3%		11.4%	88.6%		7.8%	92.2%	

In multivariable models, obese breast cancer survivors were at 2-fold higher odds (Odds Ratio [OR] 2.07, 95% CI: 1.0, 4.33) of not meeting aerobic physical activity guidelines compared to overweight or normal weight survivors. Moreover, breast cancer survivors who underwent multimodal therapy (surgery, chemotherapy, and radiation) were also at 2-fold higher odds of not meeting aerobic physical activity guidelines (OR 2.20, 95%CI: 1.17, 4.16). No other independent associations were demonstrated in models for aerobic physical activity guidelines. Regarding resistance training guidelines, breast cancer survivors with diabetes exhibited nearly 8-fold higher odds of not meeting resistance guidelines (OR 7.95, 95%CI: 1.05, 60.46); the wide confidence interval reflects a low number of women representing this category. No other independent associations were observed in models related to meeting the combination of aerobic and resistance training guidelines (Table 3).

**Table 3:** Factors associated with non-adherence to activity guideline in female BC survivors (n = 346).

Variable		Cases	Aerobic Guidelines				Resistance Guidelines				Aerobic & Resistance			
			OR	95%CI		p-level	OR	95%CI		p-level	OR	95%CI		p-level
BMI	18.5–24.99 kg/m^2^	53	1.0	reference			1.0	reference			1.0	reference		
	25–29.99 kg/m^2^	85	1.656	.755	3.633	.094	1.521	.615	3.763	.364	1.741	.648	4.676	.271
	≥ 30 kg/m^2^	208	2.077	.996	4.334	**.019**	1.889	.802	4.451	.146	2.389	.927	6.156	.071
Race/ Ethnicity	White (non-Hispanic)	207	1.0	reference			1.0	reference			1.0	reference		
	Asian	18	.919	.300	2.817	.883	.723	.216	2.418	.598	1.321	.305	5.726	.710
	African American	70	1.769	.807	3.880	.155	1.824	.630	5.283	.268	1.739	.526	5.754	.365
	Hispanic or Latino	51	1.501	.647	3.484	.344	1.373	.472	3.995	.561	1.305	.399	4.273	.660
Insurance Status	Self-Pay	11	1.0	reference			1.0	reference			1.0	reference		
	Medicare or Medicaid	114	.650	.110	3.829	.634	.761	.075	7.755	.817	.910	.083	9.912	.938
	Private Insurance	185	.662	.123	3.560	.630	.524	.058	4.747	.565	.738	.078	6.992	.791
	Not Reported	36	.523	.084	3.238	.486	.601	.055	6.518	.675	.577	.051	6.544	.657
Smoking History	Never Use	260	1.0	reference			1.0	reference			1.0	reference		
	Ever Use	86	1.240	.662	2.325	.502	1.166	.545	2.492	.692	2.081	.805	5.379	.131
Diabetes	No	285	1.0	reference			1.0	reference			1.0	reference		
	Pre-diabetes	9	3.218	.367	28.196	.291	*	*	*	*	*	*	*	*
	Yes	52	1.796	.741	4.352	.195	7.951	1.046	60.458	**.045**	6.381	0.824	49.403	.076
Menopause	Pre-menopausal	35	1.0	reference			1.0	reference			1.0	reference		
	Peri-menopausal	7	.873	.109	6.978	.898	1.701	.133	21.727	.683	1.222	.091	16.310	.880
	Post-menopausal	304	1.004	.368	2.738	.994	1.490	.444	4.998	.519	2.017	.537	7.581	.299
Multimodal Therapy	No	237	1.0	reference			1.0	reference			1.0	reference		
	Yes	109	2.204	1.167	4.161	**.015**	1.169	.562	2.430	.676	1.554	.648	3.723	.323

Adjusted for age, BMI, race/ethnicity, insurance status, cancer history, smoking history, diabetes, heart condition, chemotherapy, radiation, and surgery history* Not enough cases meeting guidelines to determine OR.

## Discussion

In the current study, we found that only 9% of cancer prevention patients and survivors referred to an exercise physiologist for consultation adhered to both aerobic and resistance physical activity guidelines. We observed that being overweight or obese was associated with whether patients were meeting guideline-based physical activity among both prevention patients and breast cancer survivors. Moreover, we observed that adjuvant therapy consisting of a combination of surgery, radiotherapy, and chemotherapy (i.e. multimodal therapy) was independently associated with poor adherence to physical activity guidelines among breast cancer survivors.

Our finding of low adherence to physical activity guidelines among cancer prevention patients and survivors is consistent with surveillance data from the Behavioral Risk Factor Surveillance System (BRFSS), National Health Interview Survey (NHIS), National Health and Nutrition Survey (NHANES), and the Surveillance, Epidemiology, and End Results Program (SEER) [4, 32, 33]. In aggregate, these data speak to the overwhelming need to provide systematic screening and interventions for patients with the goal of increasing physical activity, a key risk factor of cancer occurrence and recurrence. Refinement of health care delivery and reimbursement models are needed to encourage providers and health care systems across all clinical care settings to develop programs centered around physical activity screening and engagement.

Our role in the integrative health program within the Cancer Prevention Center at MD Anderson is to provide these types of services to patients in order to increase physical activity. We have done this by developing an algorithm that is integrated within our clinical workflow. Access to our algorithm is publicly available here: https://www.mdanderson.org/for-physicians/clinical-tools-resources/clinical-practice-algorithms.html.

Essentially, qualified clinical personnel assess the patient’s current level of activity by asking the patient about his/her exercise frequency, intensity (assessed via rating of perceived exertion of self-evaluation of exercise tolerance), duration, ad type of exercise engaged in. If the patient’s self-reported physical activity behavior meets the ACSM physical activity guidelines, then the patient is asked whether he/she is interested in increasing activity. If the patient is not, then positive reinforcement and encouragement to continue meeting guidelines is provided. If the patient is interested in increasing activity, then the patient is assessed for pre-defined (per developed algorithm) conditions that may require clearance from a medical provider. If the patient does not require medical clearance, then he/she may be referred to a clinical exercise physiologist for exercise counseling. If the patient does require medical clearance, then the patient is provided with a physical activity clearance form to be completed by her provider.

Alternatively, if the patient’s self-reported physical activity behavior is not meeting ACSM physical activity guidelines, then the patient is asked whether he/she is interested in starting or increasing activity. If the patient is not, then review of activity guidelines and motivational interviewing to encourage physical activity and limit sedentary time is provided. Additionally, an explanation of physical activity benefits for cancer prevention and a handout related to tips to getting fit is provided. Reassessment of physical activity will then occur at the next clinic visit. However, if the patient is interested in starting or increasing physical activity, then the he/she will be assessed for pre-defined conditions that may require medical clearance as described above, and this pathway in the algorithm will be followed.

Our secondary aim was to identify clinical factors associated with poor adherence to physical activity guidelines. We observed significant inverse associations between BMI and adherence to physical activity guidelines for both cancer prevention patients and breast cancer survivors. This result was largely expected, given obesity and physical inactivity are interrelated (e.g. physical inactivity may lead to weight gain, weight gain may lead to further physical inactivity, etc.). Unfortunately, obesity is an epidemic in the US population and is considered a key risk factor for 13 different types of cancer, in addition to physical inactivity [12, 34]. To this end, lifestyle programs aimed at increasing physical activity as part of weight loss efforts should be considered for both cancer prevention patients and breast cancer survivors, when appropriate.

In the current study, we found that attention to prior treatment exposures may be a way to identify breast cancer survivors who are least likely to adhere to physical activity guidelines. While breast cancer survivors who received particular adjuvant therapies (e.g. radiation, lumpectomy) were less likely to adhere to guideline-based activity compared to those not receiving such therapies, only multimodal therapy (surgery, radiation, and chemotherapy) was independently associated with poor adherence to physical activity guidelines in the current study. Our results support other work by our group demonstrating that cardiorespiratory fitness, measured via exercise treadmill testing, is significantly lower in breast cancer survivors receiving multimodal therapy compared to those receiving individual therapies alone [35, 36]. In both studies, physical activity and cardiorespiratory fitness were measured years (> 5 years on average) after the initial treatment exposure. These data reveal that low levels of physical activity or fitness are not short-term issues for breast cancer survivors receiving multimodal therapy [35, 36].

Additionally, it may also be postulated that breast cancer survivors with a history of multimodal therapy may exhibit lower adherence to physical activity guidelines and lower levels of cardiorespiratory fitness due to increased cancer-related fatigue secondary to their treatment history. A recent meta-analysis conducted by van Vulpen and colleagues [37] observed statistically significant improvements in general fatigue and physical fatigue when physical activity was conducted during adjuvant therapy among breast cancer survivors. Furthermore, a recent Cochrane systematic review conducted by Lahart and colleagues [38] observed statistically significant but smaller improvements in fatigue when physical activity was conducted post-adjuvant therapy among breast cancer survivors. Taken together, creating an infrastructure to promote and deliver exercise programs for breast cancer patients during and after multimodal therapy is needed.

Strengths of the current study include a large sample size, consideration of adherence to aerobic and resistance physical activity guidelines alone and in combination, and assessment of multimodal therapy in relation to physical activity guideline adherence. Limitations of this study include self-reported physical activity behavior, inability to examine factors associated with physical activity adherence in other cancer populations due to small sample sizes, cross-sectional design of the study, and referral to see an exercise physiologist based on provider recommendation or patient interest/inquiry. The latter may have selected for more motivated patients since some patients inquired for a referral, though our physical activity adherence percentages were in line with population-based studies. We consider the fact that our study included one-on-one counseling with a clinical exercise physiologist as both a strength and a limitation of this study. While the expertise of an exercise physiologist is certainly a benefit to our patients and to the assessment of guideline adherence, this is also a limitation as not many cancer centers provide this service and so the generalizability of our model at this time may be limited. Future research should consider targeting men and normal weight cancer prevention patients and survivors in similar analyses, assessment of objectively measured aerobic and resistance activity, assessment of other cancer types in relation to physical activity guideline adherence, and dissemination and implementation research aimed at utilizing the identified factors associated with non-adherence to physical activity guidelines to identify the most appropriate patients in need of physical activity promotion interventions.

## Conclusions

Poor adherence to physical activity guidelines among cancer prevention patients and survivors seen in a clinical cancer prevention center were similar to findings observed from surveillance and population-based cohort studies. Based on our results, a review of BMI and cancer treatment history, specifically history of multimodal therapy, should be considered in order to identify individuals at greatest risk for non-adherence to physical activity guidelines. Moreover, a greater focus on integrating physical activity as part of the conversation providers have with patients, and including activity counseling, promotion programs and resources by trained professionals into the clinical care setting will be critical in order to improve adherence to physical activity guidelines at the population level. This change will require engagement with 3^rd^ party payers regarding reimbursement of preventive services in clinical setting as well as require innovative approaches (i.e. mobile health technology) to systematically reach and promote physical activity in prevention patients and cancer survivors.

## Supporting information

S1 File Dataset. (XLSX)

## References

[1] Abioye AI, Odesanya MO, Abioye AI, and Ibrahim NA. Physical activity and risk of gastric cancer: a meta-analysis of observational studies. Br J Sports Med. 2015;49:224–29. 10.1136/bjsports-2013-092778 24434186

[2] Albanes D, Blair A, and Taylor PR. Physical activity and risk of cancer in the NHANES I population. Am J Public Health. 1989;79(6):744–50. 10.2105/ajph.79.6.744 2729471 PMC1349635

[3] McCullough ML, Patel AV, Kushi LH, Patel R, Willett WC, Doyle C, et al Following cancer prevention guidelines reduces risk of cancer, cardiovascular disease, and all-cause mortality. Cancer Epidemiol Biomarkers Prev. 2011;20(6):1089–97. 10.1158/1055-9965.EPI-10-1173 21467238

[4] McTiernan A, Kooperberg C, White E, Wilcox S, Coates R, Adams-Campbell LL, et al Recreational physical activity and the risk of breast cancer in postmenopausal women: the Women's Health Initiative Cohort Study. JAMA. 2003;290(10):1331–6. 10.1001/jama.290.10.1331 12966124

[5] Sanchis-Gomar F, Lucia A, Yvert T, Ruiz-Casado A, Pareja-Galeano H, Santos-Lozano A, et al Physical inactivity and low fitness deserve more attention to alter cancer risk and prognosis. Cancer Prev Res (Phila). 2015;8(2):105–10. 10.1158/1940-6207.CAPR-14-0320 25416409 PMC4315717

[6] Thune I, Brenn T, Lund E, and Gaard M. Physical activity and the risk of breast cancer. N Engl J Med. 1997;336(18):1269–75. 10.1056/NEJM199705013361801 9113929

[7] Thune I and Furberg AS. Physical activity and cancer risk: dose-response and cancer, all sites and site-specific. Med Sci Sports Exerc. 2001;33(6 Suppl):S530–50. 11427781 10.1097/00005768-200106001-00025

[8] Wu Y, Zhang D, and Kang S. Physical activity and risk of breast cancer: a meta-analysis of prospective studies. Cancer Res Treat. 2013;137:869–82. 10.1007/s10549-012-2396-7 23274845

[9] Farrell SW, Cortese GM, LaMonte MJ, and Blair SN. Cardiorespiratory fitness, different measures of adiposity, and cancer mortality in men. Obesity (Silver Spring). 2007;15(12):3140–9. 10.1038/oby.2007.374 18198325

[10] Inoue-Choi M, Robien K, and Lazovich D. Adherence to the WCRF/AICR guidelines for cancer prevention is associated with lower mortality among older female cancer survivors. Cancer Epidemiol Biomarkers Prev. 2013;22(5):792–802. 10.1158/1055-9965.EPI-13-0054 23462914 PMC3650116

[11] Irwin M, Smith A, McTiernan A, Ballard-Barbash R, Cronin K, Gilliland F, et al Influence of pre- and postdiagnosis physical activity on mortality in breast cancer survivors: the health, eating, activity, and lifestyle study. J Clin Oncol. 2008;26(24):3958–64. 10.1200/JCO.2007.15.9822 18711185 PMC2654316

[12] Lakoski S, Willis B, Barlow C, Leonard D, Gao A, Radford N, et al Midlife Cardiorespiratory Fitness, Incident Cancer, and Survival After Cancer in Men: The Cooper Center Longitudinal Study. JAMA Oncol. 2015;1(2):231–7. 10.1001/jamaoncol.2015.0226 26181028 PMC5635343

[13] Fong DY, Ho JW, Hui BP, Lee AM, Macfarlane DJ, Leun SS, et al Physical activity for cancer survivors: meta-analysis of randomised controlled trials. BMJ. 2012;344:e70 10.1136/bmj.e70 22294757 PMC3269661

[14] Mishra SI, Scherer RW, Geigle PM, Berlanstein DR, Topaloglu O, Gotay CC, et al Exercise interventions on health-related quality of life for cancer survivors. Cochrane Database Syst Rev. 2012;CD007566 10.1002/14651858.CD007566.pub2 22895961 PMC7387117

[15] Friedenreich CM, Wang Q, Neilson HK, Kopciuk KA, McGregor SE, and Courneya KS. Physical activity and survival after prostate cancer. Eur Urol. 2016;70(4):576–85. 10.1016/j.eururo.2015.12.032 26774959

[16] Pescatello LS, Arena R, Riebe D, and Thompson PD, eds. ACSM's Guidelines for Exercise Testing and Prescription. Ninth ed 2013, Lippincott, Williams & Wilkins: Baltimore, MD. 10.1249/JSR.0b013e31829a68cf 23851406

[17] 2018 Physical Activity Guidelines Advisory Committee Scientific Report. 2018. U.S. Department of Health and Human Services: Washington, DC.

[18] Kabat GC, Matthews CE, Kamensky V, Hollenbeck AR, and Rohan TE. Adherence to cancer prevention guidelines and cancer incidence, cancer mortality, and total mortality: a prospective cohort study. Am J Clin Nutr. 2015;101:558–69. 10.3945/ajcn.114.094854 25733641 PMC4340061

[19] Tarasenko YN, Linder DF, and Miller EA. Muscle-strengthening and aerobic activities and mortality among 3+ year cancer survivors in the U.S. Cancer Causes Control. 2018;29(4–5):475–84. 10.1007/s10552-018-1017-0 29511931

[20] Mazzilli KM, Matthews CE, Salerno EA, and Moore SC. Weight Training and Risk of 10 Common Types of Cancer. Med Sci Sports Exerc. 2019;Epub ahead of print. 10.1249/MSS.0000000000001987 30920488 PMC6697215

[21] Loprinzi P, Smit E, Lee H, Crespo C, Andersen R, and Blair SN. The "fit but fat" paradigm addressed using accelerometer-determined physical activity data. N Am J Med Sci. 2014;6(7):295–301. 10.4103/1947-2714.136901 25077076 PMC4114005

[22] Smith SG and Chagpar AB. Adherence to physical activity guidelines in breast cancer survivors. Am Surg. 2010;76(9):962–5. 20836343

[23] Crawford JJ, Holt NL, Vallance JK, and Courneya KS. A new paradigm for examining the correlates of aerobic, strength, and combined exercise: an application to gynecologic cancer survivors. Support Care Cancer. 2016;24(8):3533–41. 10.1007/s00520-016-3173-7 27021390

[24] Ottenbacher A, Yu M, Moser RP, Phillips SM, Alfano C, and Perna FM. Population Estimates of Meeting Strength Training and Aerobic Guidelines, by Gender and Cancer Survivorship Status: Findings From the Health Information National Trends Survey (HINTS). J Phys Act Health. 2015;12(5):675–9. 10.1123/jpah.2014-0003 24834485

[25] Vallerand JR, Rhodes RE, Walker GJ, and Courneya KS. Correlates of meeting the combined and independent aerobic and strength exercise guidelines in hematologic cancer survivors. Int J Behav Nutr Phys Act. 2017;14(1):44 10.1186/s12966-017-0498-7 28351397 PMC5371229

[26] Trinh L, Strom DA, Wong JN, and Courneya KS. Modality-specific exercise guidelines and quality of life in kidney cancer survivors: A cross-sectional study. Psychooncology. 2018;27(10):2419–26. 10.1002/pon.4844 30048023

[27] Forbes C, Blanchard C, Mummery W, and Courneya K. Prevalence and correlates of strength exercise among breast, prostate, and colorectal cancer survivors. Oncol Nurs Forum. 2015;42(2):118–27. 10.1188/15.ONF.42-02AP 25806879

[28] Short C, James E, Vandelanotte C, Courneya K, Duncan M, Rebar A, et al Correlates of resistance training in post-treatment breast cancer survivors. Support Care Cancer. 2014;22(10):2757–66. 10.1007/s00520-014-2273-5 24805910

[29] Fisher A, Williams K, Beeken R, and Wardle J. Recall of physical activity advice was associated with higher levels of physical activity in colorectal cancer patients. BMJ Open. 2015;5(4):e006853 10.1136/bmjopen-2014-006853 25922098 PMC4420935

[30] Jones LW, Courneya KS, Fairey AS, and Mackey JR. Effects of an oncologist's recommendation to exercise on self-reported exercise behavior in newly diagnosed breast cancer survivors: a single-blind, randomized controlled trial. Ann Behav Med. 2004;28(2):105–13. 10.1207/s15324796abm2802_5 15454357

[31] Tarasenko Y, Miller E, Chen C, and Schoenberg N. Physical activity levels and counseling by health care providers in cancer survivors. Prev Med. 2017;99:211–17. 10.1016/j.ypmed.2017.01.010 28131780

[32] Irwin ML, McTiernan A, Bernstein L, Gilliland FD, Baumgartner R, Baumgartner K, et al Physical activity levels among breast cancer survivors. Med Sci Sports Exerc. 2004;36(9):1484–91. 15354027 PMC3000611

[33] Nayak P, Holmes HM, Nguyen HT, and Elting LS. Self-reported physical activity among middle-aged cancer survivors in the United States: Behavioral Risk Factor Surveillance System Survey, 2009. Prev Chronic Dis. 2014;11:E156 10.5888/pcd11.140067 25211504 PMC4165550

[34] Lauby-Secretan B, Scoccianti C, Loomis D, Grosse Y, Bianchini F, and Straif K. Body fatness and cancer- viewpoint of the IARC working group. N Engl J Med. 2016;375(8):794–8. 10.1056/NEJMsr1606602 27557308 PMC6754861

[35] Lakoski SG, Barlow CE, Koelwyn GJ, Hornsby WE, Hernandez J, Defina LF, et al The influence of adjuvant therapy on cardiorespiratory fitness in early-stage breast cancer seven years after diagnosis: the Cooper Center Longitudinal Study. Breast Cancer Res Treat. 2013;138(3):909–16. 10.1007/s10549-013-2478-1 23504137 PMC3640334

[36] Peel AB, Thomas SM, Dittus K, Jones LW, and Lakoski SG. Cardiorespiratory fitness in breast cancer patients: a call for normative values. J Am Heart Assoc. 2014;3(1):e000432 10.1161/JAHA.113.000432 24419734 PMC3959685

[37] van Vulpen JK, Peeters PH, Velthuis MJ, van der Wall E, and May AM. Effects of physical exercise during adjuvant breast cancer treatment on physical and psychosocial dimensions of cancer-related fatigue: A meta-analysis. Maturitas. 2016;85:104–11. 10.1016/j.maturitas.2015.12.007 26857888

[38] Lahart IM, Metsios GS, Nevill AM, and Carmichael AR. Physical activity for women with breast cancer after adjuvant therapy. Cochrane Database Syst Rev. 2018;CD011292 10.1002/14651858.CD011292.pub2 29376559 PMC6491330

